# Trace Amounts of Furan-2-Carboxylic Acids Determine the Quality of Solid Agar Plates for Bacterial Culture

**DOI:** 10.1371/journal.pone.0041142

**Published:** 2012-07-27

**Authors:** Shintaro Hara, Reika Isoda, Teemu Tahvanainen, Yasuyuki Hashidoko

**Affiliations:** 1 Graduate School of Agriculture and Research Faculty of Agriculture, Hokkaido University, Sapporo, Japan; 2 Department of Biology, University of Eastern Finland, Joensuu, Finland; Arizona State University, United States of America

## Abstract

**Background:**

Many investigators have recognised that a significant proportion of environmental bacteria exist in a viable but non-culturable state on agar plates, and some researchers have also noticed that some of such bacteria clearly recover their growth on matrices other than agar. However, the reason why agar is unsuitable for the growth of some bacteria has not been addressed.

**Methodology/Principal Findings:**

According to the guide of a bioassay for swarming inhibition, we identified 5-hydroxymethylfuran-2-carboxylic acid (5-HMFA) and furan-2-carboxylic acid (FA) as factors that inhibit bacterial swarming and likely inhibit extracellular polysaccharide production on agar. The furan-2-carboxylic acids 5-HMFA and FA effectively inhibited the swarming and swimming of several environmental bacteria at concentrations of 1.8 and 2.3 µg L^−1^ (13 and 21 nmol L^−1^), respectively, which are equivalent to the concentrations of these compounds in 0.3% agar. On Luria-Bertani (LB) plates containing 1.0% agar that had been previously washed with MeOH, a mixture of 5-HMFA and FA in amounts equivalent to their original concentrations in the unwashed agar repressed the swarming of *Escherichia coli* K12 strain W3110, a representative swarming bacterium.

**Conclusions/Significance:**

Agar that contains trace amounts of 5-HMFA and FA inhibits the proliferation of some slow-growing or difficult-to-culture bacteria on the plates, but it is useful for single colony isolation due to the ease of identification of swarmable bacteria as the non-swarmed colonies.

## Introduction

Agar is an indigestible gelling material that is derived from seaweeds such as *Gelidium crinale* and *Gracilaria vermiculophylla.* Since the dawn of bacteriology in the late 19^th^ century, bacteriologists have used agar as a matrix for plate culture and as soft gel media, as it is relatively inexpensive and easy to handle [1]–[3]. However, environmental bacteria often do not thrive on agar media, even when provided with adequate oxygen and other requirements. Over 95% of environmental bacteria from the ocean, soil, lake sediments, river water, plant surfaces or the interior walls of animal intestines grow slowly or are difficult to culture; these bacteria are referred to as being viable but non-culturable (VBNC) [4]–[8]. Despite many efforts to improve culture conditions by altering nutrient components, pH or other physicochemical conditions, few breakthroughs have been achieved in culturing VBNC bacteria to date [1], [9], [10].

**Figure 1:**
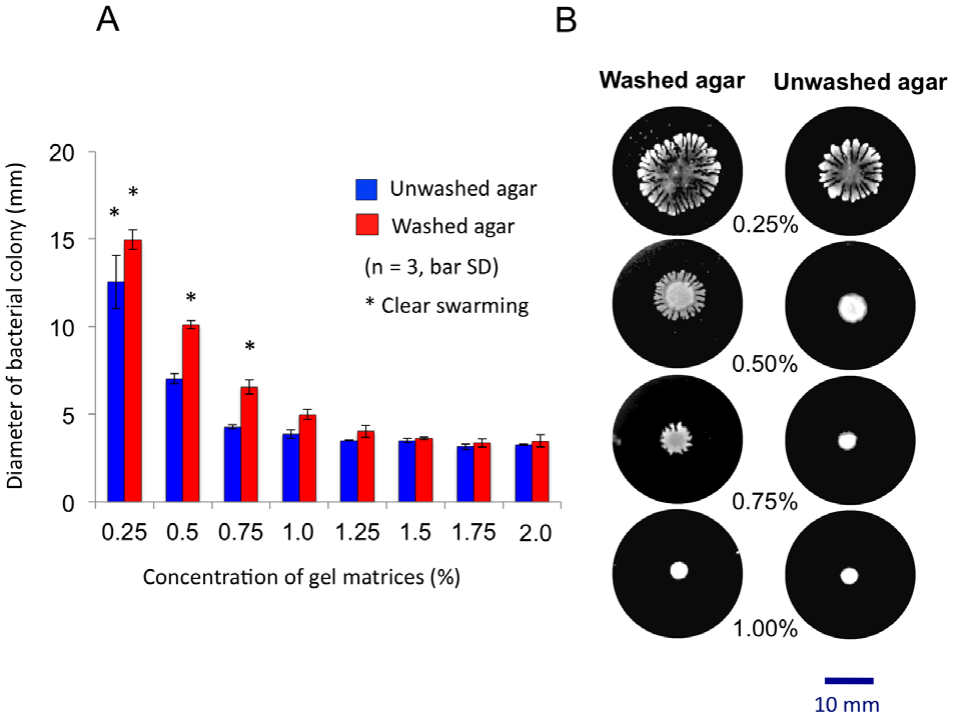
*Pseudomonas collierea* V5-G’5 swarming and swimming on plates solidified with unwashed agar or solvent-washed agar. (A) The swarming and swimming behaviours of *Pseudomonas collierea* V5-G’5 were tested on MW plates containing unwashed agar or EtOAc/MeOH-washed agar (washed agar). The maximum diameter of each colony after a 36-h incubation at 20°C was measured with an electric micrometer caliper. Bar, ± standard deviation (SD) (n = 3). (B) Images of swarmed *P. collierea* V5-G’5 colonies on the assay plates containing washed (left) and unwashed (right) agar, after a 36-h incubation.

Some studies have found that replacing agar with gellan gum in solid media accelerates the growth of slow-growing bacteria [11]–[15], particularly those from soil or rhizosphere that utilize only extracellular polysaccharides, xanthan and gellan as their carbon source [14], [16]. We have also found that some slow-growing soil bacteria in the soil bed from a boreal larch forest generated minute colonies on 1.0% agar plates containing modified Winogradsky’s (MW) medium, although they proliferated well and formed swarmed colonies on 1.0% gellan plates in MW [17]. *Pseudomonas collierea* V5-G’5 isolated from isolated from an East Siberian larch forest bed soil exhibited clearly distinguishable behaviours on the plates of agar and gellan. Conversely, MW plates containing 1.0 or 1.5% gellan gum that had been previously washed with MeOH (washed gellan) slightly repressed the swarming of *P. collierea* V5-G’5 [17]. In contrast, the swarming or swimming of *P. collierea* V5-G’5 was completely abolished on 0.5 to 0.7% unwashed agar in the same medium despite high wettability (Fig. 1A) [18], whereas medium containing 0.5% or even 0.75% agar that had previously been washed with MeOH (washed agar) allowed the bacterial cells to swarm (Fig. 1B).

Although MW plates containing 0.25 to 2.0% MeOH-washed gellan gum slightly repressed V5-G’5 swarming, *P. collierea* V5-G’5 still formed swarmed colonies that were clearly larger than those on MW plates with the same concentrations of washed agar. In addition, 10-fold higher concentrations of gellan gum extracts did not induce V5-G’5 swarming on plates containing 0.5 to1.5% unwashed agar. We therefore hypothesised that MeOH-soluble chemical constituents in agar are the primary factors that inhibit swarming of *P. collierea* V5-G’5 on agar plates, as reported previously in some studies on swarming inhibitors [19], [20].

## Results

### Isolation and Identification of Swarming-inhibiting Principles from Powdered Agar

**Figure 2:**
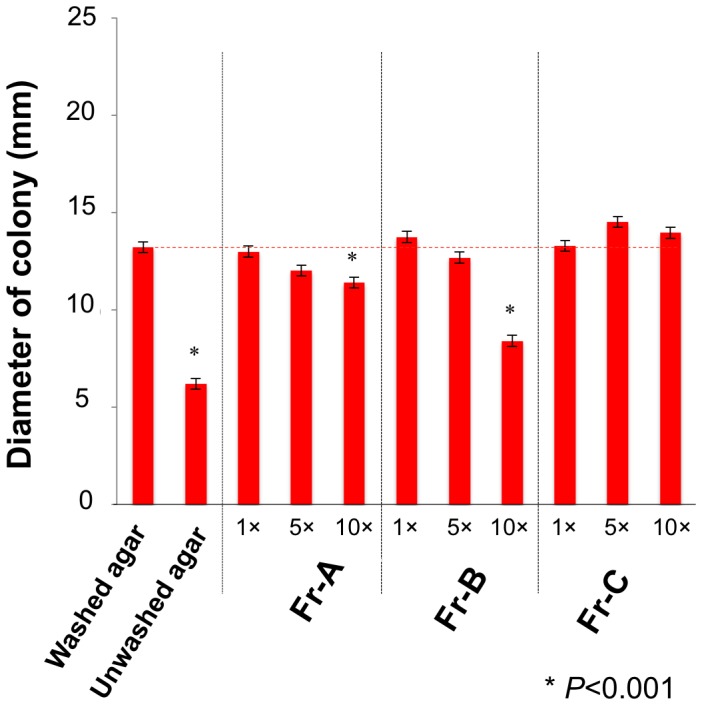
Swarming and swimming inhibition by the active fractions of 70% EtOAc/MeOH agar extracts. The EtOAc/MeOH extracts of powdered agar dissolved in 100 µL Milli-Q water were dissolved in 20 mL of the washed agar medium before autoclaving. Both Fr-A and Fr-B that inhibited swarming of *P. collierea* V5-G’ 5 on washed agar and Fr-C inactive as eluted with 40 to 50% MeOH/CHCl_3_ were bioassayed at concentrations equivalent to those found in 3.0% gels. Bar, ± SD (n = 3). * *P*<0.001 compared with the washed agar (left) using the Student’s *t*-test.

Using the bioassay for swarming inhibition of *Pseudomonas collierea* V5-G’5, the bioassay-guided fractionation of the low molecular weight, active principles was investigated in 70% EtOAc/MeOH extracts (1.46 g) prepared from 5 kg agar powders (Wako, Osaka, Japan). The active fractions Fr-A and Fr-B (17.3 and 7.7 mg respectively) were obtained as by reverse-phase column chromatography and silica gel column chromatography eluted with water and CHCl_3_-MeOH-H_2_O (65∶25: 4) respectively (Scheme S1). Both fractions inhibited swarming of *P. collierea* V5-G’5 on washed agar at concentrations equivalent to that found in 3.0% gels (Fig. 2).

**Figure 3:**
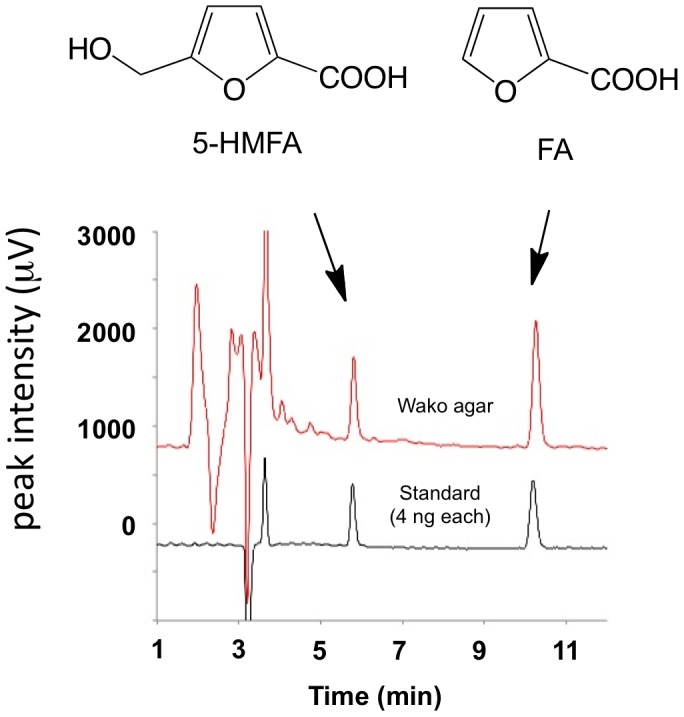
HPLC profile and quantification of 5-HMFA and FA in several powdered agars and gellan. HPLC profiles for 5-HMFA and FA in agar (*tR* 5.7 min and 9.2 min respectively) shown in this figure were those performed on an analytical column of L-column2 ODS (4.6×150 mm). In the semi-preparation column (10×250 mm) for practical purification of 5-HMFA and FA, retention times of peaks 1 and 2 were relatively longer (*tR* 8.8 and 15.4 min respectively) than those in the analytical column.

The active fraction Fr-B processed by reverse-phase HPLC using 10% CH_3_CN/H_2_O containing 0.2% HCOOH yielded peak 1 at *tR* 5.7 min (1 mg). In Fr-A, peak 1 (1 mg) and peak 2 at *tR* 9.2 min (1 mg) were obtained as the major substances (Fig. 3). Peak 1 from both fractions Fr-A and Fr-B was identical to 5-hydroxymethylfuran-2-carboxylic acid (5-HMFA) using FD-MS, EI-HR-MS and ^1^H NMR [21], [22]. The physicochemical properties of the active substance agreed with the synthetic 5-HMFA, which was derived from ethyl 5-chloromethyl-2-furancarboxylate in two steps (85% total yield) (Scheme S2). Peak 2 in Fr-A was identified as furan-2-carboxylic acid.

### Quantitative Analysis of 5-HMFA and FA in Dry Agar Products

MeOH extracts from a 5.00 g-scale of Wako agar powders generally used in this study were analyzed by HPLC after some clean-up processes as follows. The agar powder (5.00 g) was mixed with 25 mL of MeOH in 50-mL Falcon tubes, sonicated for 30 min, and centrifuged (3,500×*g*). Next, 20 mL of the MeOH extract was recovered by filtration from the supernatant. This process was repeated three times for a calculated recovery of 99.5%. All MeOH extracts were combined, concentrated *in vacuo*, and dissolved in 1 mL of Milli-Q water. The aqueous extract was filtered through 0.45-µm PTFE membrane cartridges (Advantec, Tokyo, Japan) and directly applied to a Sep-Pak C18 column (Varian, Palo Alto, CA). The inner lines including the column were washed with Milli-Q water to collect all filtered extracts into 5-mL volumetric flasks. Small portions were then filtered with a Millex®-LG filter unit (Millipore, 0.2 µm PTFE), and 10-µL samples were analyzed by HPLC and monitored by UV absorbance at 245 nm. The quantitative analysis indicated 5-HMFA and FA yields of 0.58±0.04 and 0.75±0.00 µg g^−1^ (4.1 and 6.7 nmol g^−1^) from Wako agar powders, respectively. Conversely, the MeOH-washed powdered agar, which was prepared from the same Wako agar, contained 0.07 and 0.12 µg g^−1^ (0.5 and 1.1 nmol g^−1^) of 5-HMFA and FA, respectively, indicating that the extraction had removed 85 to 90% of the compounds.

**Figure 4:**
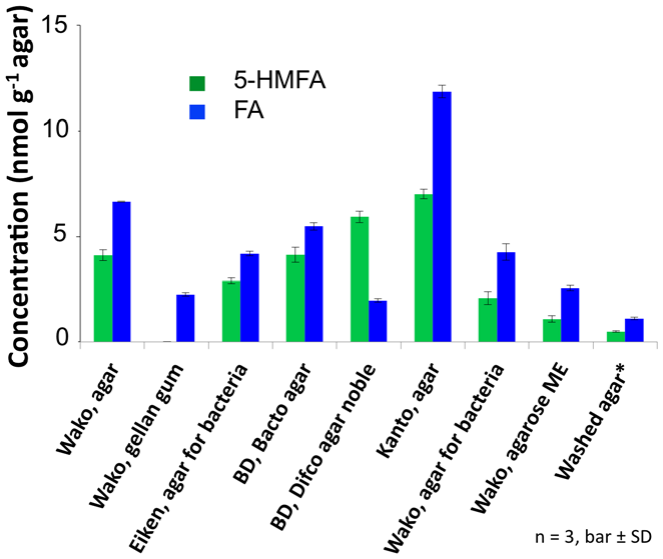
Contents of 5-HMFA and FA in powdered agar products from different companies and powdered gellan. Concentrations of 5-HMFA and FA in some commercially available agar powders, products of several different companies, were quantitatively analyzed by HPLC. In parallel, gellan gum (Wako) and the residual agar powder from the large-scale MeOH extraction used to generate the washed agar were also analyzed by our quantification system. All the quantitative analyses were performed in triplicate (three tubes per sample). Bar, ± SD (n = 3). Calibration curves of 5-HMFA and FA are shown in Supplementary Fig. S1.

Concentrations of 5-HMFA and FA in powdered agar products from different companies and powdered gellan were also analyzed. Eiken agar (Eiken Chemical, Tokyo, Japan) and Difco swarming agar (DB, Franklin Lakes, NJ, USA), both of which are often used for swarming studies of bacteria due to their accelerating effects on bacterial swarming [23], [24], contained relatively lower concentrations of the furan-2-carboxylic acids than those in the unwashed Wako agar (Fig. 4). We detected FA in the extracts of powdered gellan gum at approximately one-third of the concentration found in Wako agar but did not detect 5-HMFA.

### Swarming and Swimming Inhibiting Activities of Synthesized 5-HMFA and FA on Some Bacteria

**Figure 5:**
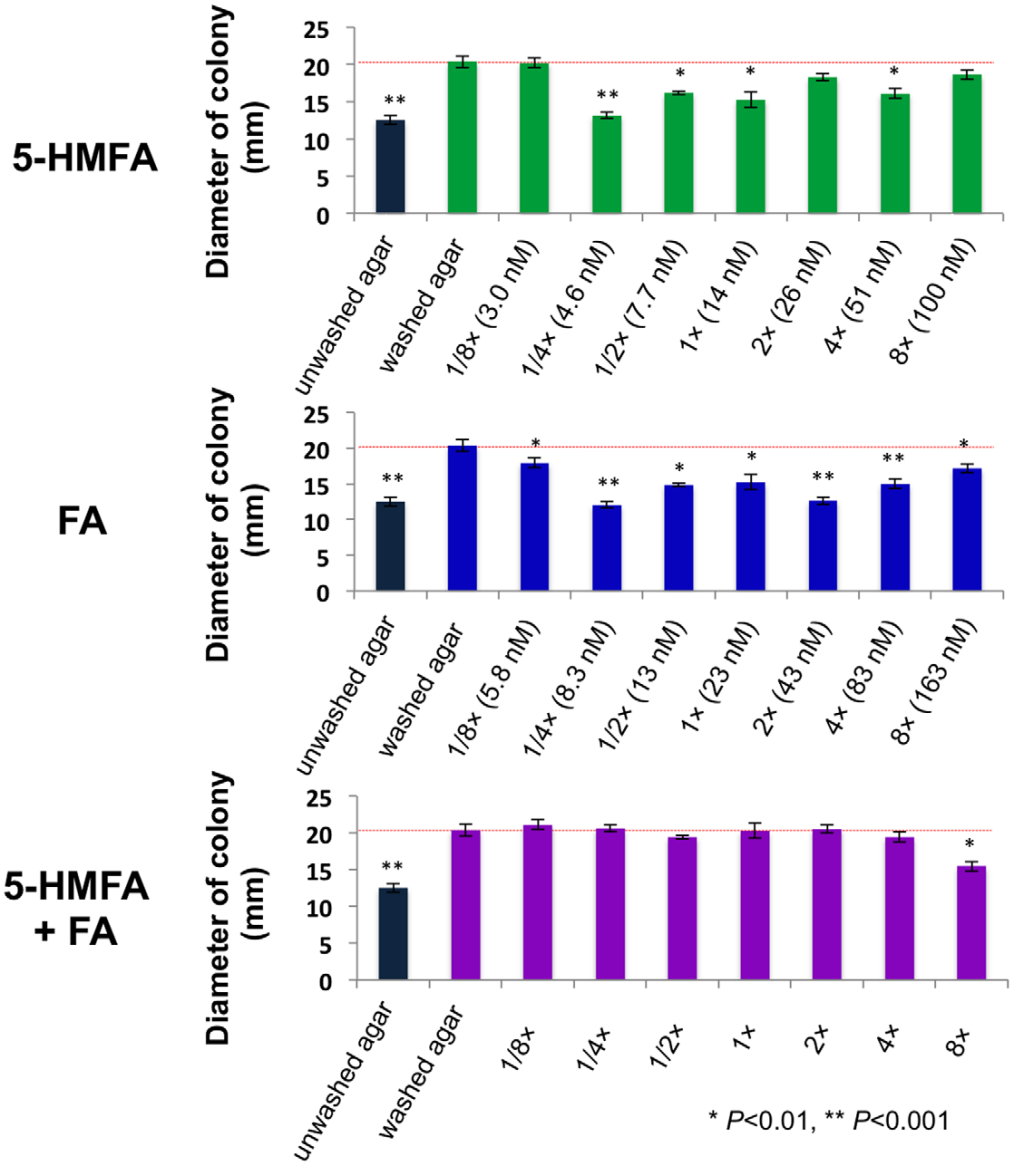
Swarming repressing activities of 5-HMFA and FA toward *Pseudomonas collierea* V5-G’5. Supplemented amounts of furan-2-carboxylates were calculated in original concentrations in 0.3% unwashed agar powders, without any consideration of residuals in the washed agar. Both 5-HMFA and FA showed significant inhibition against *P. collierea* V5-G’5 swarming with good reproducibility, whereas the mixture of the furan-2-carboxylates (a molar ratio of 13∶21 for 5-HMFA and FA) showed inhibition at those equivalent to 2.4% untreated agar.

Bioassay for swarming and swimming of *P. collierea* V5-G’5 on MW plates containing 0.3% washed agar showed that supplemented 13 nmol L^−1^ 5-HMFA or 21 nmol L^−1^ FA affected significant inhibition of its swarming with high reproducibility. Conversely, a mixture of the furan-2-carboxylic acids (at the molar ratio of 13∶21 of 5-HMFA and FA) showed inhibition at levels equivalent to 2.4% unwashed agar (Fig. 5).

**Figure 6:**
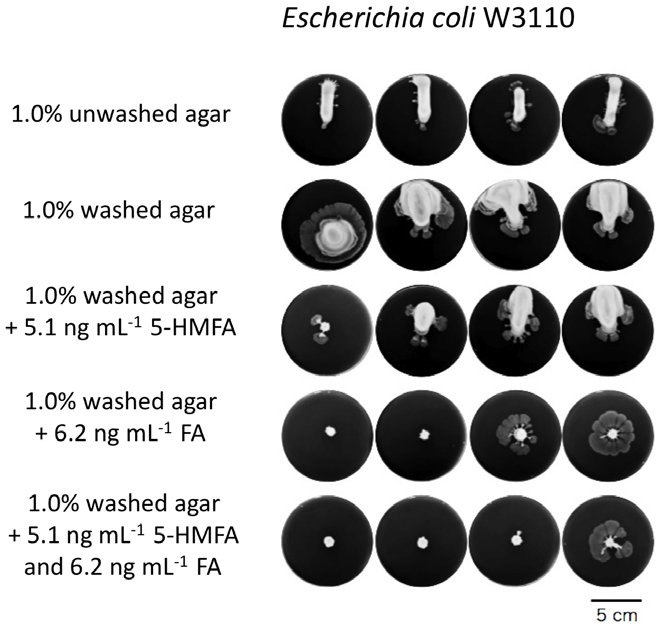
Swarming repression of swarmable *Escherichia coli* on Luria-Bertani (LB) agar plates containing 5-HMFA and FA. Although the LB plates containing 1.0% unwashed agar allowed the swarmable *E. coli* K12 strain W3110 formation of a fluidized colony, whereas those containing 1.0% washed agar induced active swarming to the strain. Swarming suppressing activity of FA on LB plates that contained 1.0% washed agar was clearly observed at the original concentration of FA in 1.0% unwashed agar. The washed agar also lost the ability to form a fluidized colony as observed on the 1.0% unwashed agar-containing LB plates. On 0.6% gel matrices, any treatments did not halt the swarming of strain W3110.

We examined whether media solidified with the washed agar with or without supplemental 5-HMFA and FA at their original concentrations in agar affect on bacterial cell growth, behaviour and physiological responses using the swarming strain *Escherichia coli* K12 strain W3110 [25]. This strain swarmed at the initial position of a colony on Luria-Bertani (LB) plates containing 1.0% washed agar, but strain formed a discrete colony that did not swarm on the same LB plates with 1.0% unwashed agar. LB plates containing washed agar and supplemented with the original amounts of furan-2-carboxylates clearly suppressed both the swarming and fluidization of the colony (Fig. 6).

### Repression of Bacterial Swimming by Furan-2-carboxylates

**Figure 7:**
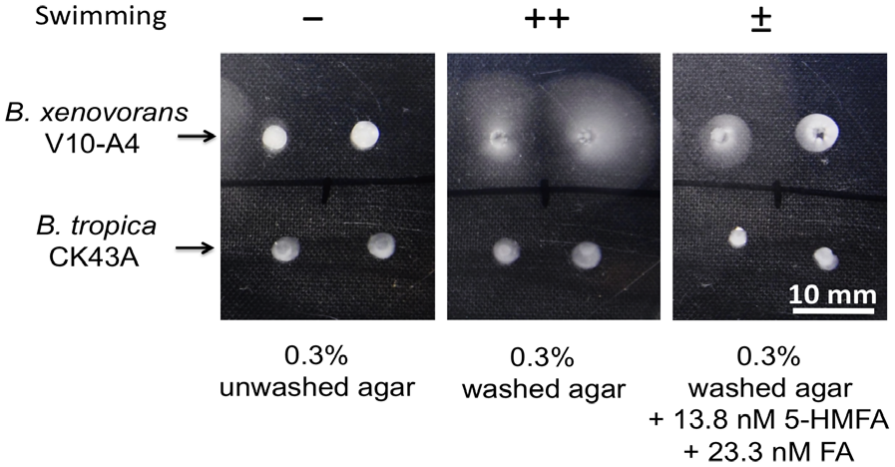
Repression of bacterial swimming by a combination of the furan-2-carboxylates. On 0.3% agar plates of MW medium, a swarmable β-proteobacterium *Burkholderia xenovorans* V10-A4 was inoculated as an aliquot (1 µL) of bacterial cell suspension as 2×10^1^ (upper, left colony) and 2×10^0^ cells (upper, right colony). Dual cultured *Burkholderia tropica* CK43A (lower colonies, ca. 1×10^3^ cells) was seeded next to the *B. xenovorans* V10-A4 at a distance of 13 to 16 mm. On the plate containing 0.3% washed agar, *B. xenovorans* V10-A4 actively swam in the soft gel (center), while the same plate supplemented with a 5-HMFA and FA mixture equivalent to that contained in 0.3% unwashed agar cancelled the swimming of *B. xenovorans* V10-A4 (right). Swimming in the 0.3% washed agar gel was observed within 24 h.

The supplemented furan-2-carboxylic acids mixture also inhibited swimming behaviour of a Siberian swarming soil bacterium, *Burkholderia xenovorans* V10-A4 (Fig. 7). After a 1-µL aliquot of *Burkholderia tropica* CK43A cell suspension (1×10^3^ cells) was inoculated as a swimming stimulant on MW containing 0.3% washed agar, also a 1-µL aliquot of *B. xenovorans* V10-A4 cell suspension was point inoculated next to the *B. tropica* CK43A at a distance of 12 mm as 2×10^1^ and 2×10^0^ cells. Subsequently, *B. xenovorans* V10-A4 cell exhibited active swimming in the soft gel within 24 h, but did not show any swimming behaviour on MW plates containing 0.3% unwashed agar. Plates containing 0.3% washed agar and supplemented with a mixture of the furan-2-carboxylic acids equivalent to the original concentrations in unwashed agar (13 and 20 nmol L^−1^ of 5-HMFA and FA) also completely abolished the swimming behaviour of *B. xenovorans* V10-A4. This swimming behaviour observed to be a result of a direct interaction between *B. xenovorans* V10-A4 and *B. tropica* CK43A was inhibited by the furan-2-carboxylic acids as its mediators.

### Effective Proliferation of a Slow-growing Bacterium as Swarmed Colonies on 1.5% Washed Agar Plates

**Figure 8:**
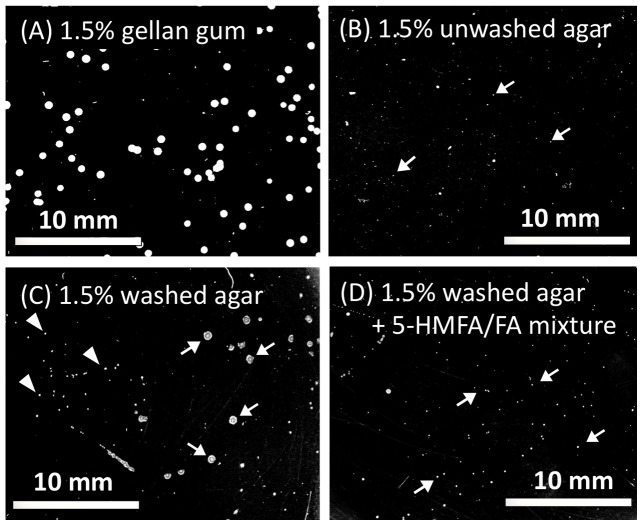
Proliferation of a slow-growing bacterium *Cryocola antiquus* as swarmed colonies on 1.5% washer agar plate. A slow-growing actinobacterium, *C. antiquus*, formed a half-transparent, domal colony as 0.4 to 0.6 mm in diameter on 1.5% gellan plates of Winogradsky’s mineral solution supplemented with the 0.05% carbon source mixture (A). On the same plates except for 1.5% washed agar instead of gellan gum, *Cryocola antiquus* appeared as a swarmed, flat colony as 0.5 to 0.6 mm in diameter (C). In some area where the bacteria were seeded as relatively high density, the colony size was less than 0.1 mm (area in the left). Plates with 1.5% unwashed agar (B) and 1.5% washed agar supplemented with the mixture of 5-HMFA and FA (D) showed the tiny colonies of 0.03 mm in the diameter. All the plates were incubated at 20°C for 3 weeks.

On the 1.5% washed agar plates that were supplemented with 0.05% of the carbon source mixture while without any nitrogen sources, a slow-growing actinobacterium *Cryocola antiquus* isolated from a subarctic tundra soil in Finland showed slight swarming on the plate, and sufficient proliferation of *C. antiquus* occurred to form visible colonies (C in Fig. 8). Conversely, the small amounts of the mixture of furan-2-carboxylic acids in the agar repressed the swarming behaviour, resulting in tiny colonies (less than 0.01 mm in the diameter) hardly visible without mesoscopic observation even after 3-week incubation (D).

## Discussion

### Significant Roles of the Furan-2-carboxylates

At the original or lower concentrations, both 5-HMFA and FA showed statistically significant (Student’s *t*-test distribution, *P*<0.001 and *P*<0.01) swarming repression, but the inhibitory effects were not dose-dependent, similar to some bacteria to quormone mimics [26]. Also, an excessively high concentration of 5-HMFA or FA (*e.g*. 100 mg L^−1^) did not inhibit the cell growth of the bacteria tested. In contrast, a mixture of these furan-2-carboxylic acids at a ratio equivalent to their original concentrations in powdered agar showed swarming inhibition (*P*<0.01) only at 8×concentrations (equivalent to 2.4% agar).

Most bacteriologists over approximately the past century have used agar containing the furan-2-carboxylic acids as the gelling material for plate cultures. On these plates, many bacteria grow as individual colonies of a manageable size that do not overwhelm neighbouring colonies due to swarming or swimming suppression by the furan-2-carboxylic acids in processed agar (Figs. 6 and 7). However, these properties also result in agar being an inappropriate solid medium for studying most slow-growing, oligotrophic environmental bacteria (Fig. 8). The presence of 5-HMFA, FA and potentially other unknown compounds in agar may lead to in many bacteria assuming the VBNC or slow-growing state, whereas gellan gum, which has no 5-HMFA, often allows the proliferation of some VBNC bacteria regardless of nutritional demands [27], [28]. We also consider the presence of some quormone mimic(s) or surfactant(s) as a fluidising component in the gellan gum [29], [30], [31], probably allowing the bacterial swarming on gellan plates to be visible as a single colony. The identification of these important functions of 5-HMFA and FA could be a significant breakthrough to improve bacterial culture.

Inhibiting this bacterial behaviour on solid medium by adding extremely low concentrations of 5-HMFA and FA would be of great benefit for the study of many species of bacteria that are grown on solid agar media. Recent advances in understanding the mechanisms of how swarming and swimming are regulated have shown that many bacteria share systems for regulating these behaviours [23], [29], [32]. The discovery of the swarming-repressing activity of the furan-2-carboxylates that occur at extremely low concentrations in agar, therefore, may lead to a breakthrough in plate culture technology for several bacteria that are currently difficult to culture on agar plates.

### Possible Source of 5-HMFA in Agar

Reports describe 5-HMFA as a common natural product that is a major by-product of fermented liquid sugar [33], [34], and a major metabolite of some basidiomycete and ascomycete fungi [35], [36]. It is also a metabolite of the endophytic fungus *Apiospora montagnei*, which is found in brown seaweed [35]. Therefore, some brown or red algae used to make agar could be contaminated with the endophytic fungi that produce 5-HMFA. In contrast, it is possible that 5-HMFA is an artefact of the production of powdered agar, considering that heating sugars spontaneously generates 5-HMFA [34]. FA is also widely found in fermented sugars, cooked foods, etc.

## Materials and Methods

### Bioassay

The EtOAc/MeOH extracts of powdered agar dissolved in 100 µL Milli-Q water were dissolved in 20 mL of the washed agar medium before autoclaving. The final concentrations of the additives were equivalent to the ratio recoverable from unwashed agar gel matrices in the original assay plates. Liquefied medium containing the gel matrices (4.5 mL) was poured into 3.5-mm wells in 6-well plates and allowed to solidify in a sterile cabinet for 10 min. A suspension (1 µL) of *Pseudomonas collierea* V5-G’5 was point-inoculated onto the centre of each well. The swarming and swimming responses of *P. collierea* V5-G’5 were analysed to assess swarming inhibition by the active compounds of agar using several chromatographic fractions.

*P. collierea* V5-G’5 used in the bioassay for swarming repression was pre-cultured in Winogradsky’s medium containing 10 g L^−1^ sucrose and 50 mg L^−1^ yeast extract with shaking at 120 rpm at 25°C for 24 h and stored at −80°C as 10% glycerol stocks. The culture stock defrost was directly used as the inoculant. An aliquot (1 µL) of the stock-culture was inoculated onto the center of each 35-mm well in six-well plates (Nalgene, NY, USA) that had previously been filled with 4.5 mL of media solidified with 0.3% agar.

### Isolation of Active Substances

Commercial agar powders (5 kg; Wako, Osaka, Japan) were extracted with 20 L of 70% EtOAc/MeOH followed by 20 L of 100% MeOH. For the bioassay-guided isolation of the swarming repressing substances, 1.46 g of the 70% EtOAc/MeOH extract was chromatographed in a reverse-phase column (Cosmosil 75C18-OPN, Nacalai Tesque, Tokyo, Japan) conditioned with water, and the column was first eluted with water followed by MeOH containing water. The activity was observed only in the fraction eluted with water (159 mg of colorless solids). Next, the solid obtained from the active fraction via evaporation to dryness was re-suspended into an 100 mL MeOH and sonicated for some minutes, and the insolubles were removed by a Kiriyama-type Büchner funnel. The MeOH soluble substances were re-dissolved in CHCl_3_-MeOH (7∶3), and separated solubles and insoluble in the same process. The swarming repressing activity was detected only in the CHCl_3_-MeOH solubles (53 mg). The active substances re-dissolved in MeOH were further fractionated by chromatography in a silica gel column conditioned and eluted with CHCl_3_-MeOH-H_2_O (65∶25: 4). The active fractions Fr-A and Fr-B (17.3 and 7.7 mg, respectively) were eluted with 10 to 20% and 30% MeOH/CHCl_3_ followed by non-active Fr-C with 30 to 50% MeOH/CHCl_3_ (13.3 mg) (Fig. 2). Fractions Fr-A and Fr-B inhibited the swarming of *P. collierea* V5-G’5 at 10 µg L^−1^ and 4 µg L^−1^, respectively, which are concentrations that are equivalent to the amounts typically found in 3% agar) (Fig. 2). In contrast, Fr-C had no swarming inhibiting activity at the concentration equivalent to 3% agar (10×).

The active fraction Fr-B yielded one UV-quenching spot at *R_f_* 0.10 on a silica gel F_254_ TLC plate (Merck, Darmstadt, Germany) using CHCl_3_-MeOH-H_2_O (65∶25: 4), whereas Fr-A yielded two spots at *R_f_* 0.15 and 0.10. Subsequently, Fr-B was processed by reverse-phase HPLC (L-column2 ODS, 10×250 mm for semi-preparation, Chemicals Evaluation and Research Institution, Tokyo, Japan) using 10% CH_3_CN/H_2_O containing 0.2% HCOOH as the mobile phase with a flow rate of 1 mL min^−1^ for 0 to 20 min. Detection at 245 nm yielded peak 1 (*tR* 8.8 min, 1 mg of a colourless syrup) in Fr-B. In Fr-A, peak 1 (1 mg) and peak 2 (*tR* 15.4 min, 1 mg) were the major substances (Fig. 3). These predicted active factors were further isolated from the 100% MeOH extract (1.92 g), ultimately yielding 2 and 3 mg, respectively.

### Swarming Repression Assay for Furan-2-carboxylic Acids

The inhibitory effects of 5-HMFA, FA, and a mixture of the furan-2-carboxylic acids on the swarming and swimming of *P. collierea* V5-G’5 were assayed on MW solid medium (pH 6.0) containing 0.3% washed agar. The concentrations of these compounds were determined by the concentrations found in 0.3% agar plates (1×), and a series of two fold dilutions ranging from 0.125× to 8× was tested. As 5-HMFA and FA remained in the washed agar at concentrations of 0.07 and 0.12 µg g^−1^ agar, respectively, the 0.3% agar medium supplemented with 0.125× furan-2-carboxylic acids, contained 0.32 ng mL^−1^ and 0.51 ng mL^−1^ FA, respectively.

### Swarming Repression Assay on K12 Strain W3110

The solid medium used in the swarming assay for *E. coli* K12 strain W3110 (IFO 12713) was Luria-Bertani (LB) medium (10 g Bacto tryptone, 5 g Bacto yeast extract, 10 g NaCl, 5 g D-glucose per litre) solidified with 10 g L^−1^ agar. Alternative powdered agar included commercial agar (Wako), our washed agar, or washed agar supplemented with 5-HMFA, FA, or a mixture of the furan-2-carboxylates in amounts equivalent to those typically found in 10 g L^−1^ agar. The supplemented furan-2-carboxylates (36 nmol L^−1^ and 56 nmol L^−1^ of 5-HMFA and FA, respectively) were precisely adjusted to the original concentration, taking into account the small amounts that remained in the washed agar. A 2-µL *E. coli* K12 strain W3110 cell suspension prepared from colonies pre-cultured on an LB agar plate was inoculated onto the centre of a 15-mL swarming assay plate, and the inoculate was kept for 10 min in a sterile chamber with the lid removed to eliminate all the remaining water dew or aqueous film on the surface of gel plate. The plates were incubated at 30°C for 21 h.

### Chemical Derivatization of 5-HMFA from Ethyl 5-chloromethyl-2- furancarboxylate

The starting material (ethyl 5-chloromethyl-2-furancarboxylate, 1.96 g, Tokyo Chemical Industry Ltd., Tokyo, Japan) was refluxed in MeCN for 3 h with 2 eq. of AcOK and 0.1 eq. of 18-C-6 as a phase-transfer catalyst to yield ethyl 5-acetoxymethyl-2-furancarboxylate as pale brown oil (2.12 g, 96%). Pure ethyl 5-acetoxymethyl-2-furancarboxylate (1.83 g) was moderately hydrolysed with 20 eq. NaOH in 28 mL of H_2_O-EtOH (5∶2) at room temperature for 2 h. After evaporation, the aqueous reaction mixture was acidified with excess HCl, diluted with saturated NaCl to approximately 50 mL and extracted twice with an equal volume of EtOAc. The organic layers were dried over anhydrous MgSO_4_ then concentrated *in vacuo*. The resulting 5-HMFA, which resolved as a single spot on TLC, was crystallized in MeOH/CHCl_3_ (1∶9) to yield 1.09 g of needles (85% total yield) (Scheme S1). The crude crystals were re-crystallized in EtOH as fine, colorless columns, which were used for the calibration curve and bioassays.

Physicochemical properties of the furan-2-carboxylates are as follows: 5-hydroxymethylfuran-2-carboxylic acid (5-HMFA) was isolated as a colorless solid (2 mg) using HPLC with the following measures. ESI-MS (rel. int. %, positive ion mode): *m/z* 142 (M^+^, 50%), 121 (100%). EI-HR-MS: found 142.0260 (calcd. 142.0266, C_6_H_6_O_4_). ^1^H NMR (δ_H_, acetone-*d*_6_): 7.15 (d, *J* = 3.3 Hz, 1H), 6.50 (d, *J* = 3.3 Hz, 1H), and 4.58 (s, 2H). Furan-2-carboxylate (FA, 3 mg) was also isolated as a colorless solid using HPLC with the following measures: Furan-2-carboxylic acid (as ammonium salt, 3 mg). EI-MS (rel. int. %): *m/z* 128 (M^+^+NH_2_, trace), 112 (M^+^, 40%), 95 (34%), 44 (100%); EI-HR-MS: found 112.0141 (calcd. 112.0160, C_5_H_4_O_2_); and ^1^H NMR (δ_H_, CD_3_OD): 7.51 (dd, *J* = 3.3 and 1.7 Hz, 1H), 6.92 (dd, *J* = 3.3 and 0.7 Hz, 1H), 6.44 (dd, *J* = 3.3 and 1.7 Hz, 1H).

### Quantitative Analysis of 5-HMFA and FA

We prepared calibration curves for the synthetic or authentic 5-HMFA and FA, both of which are water soluble, for the quantitative HPLC analysis of the experimental gel matrices (Fig. S1). The correlation coefficients of the standard curves were *r* ^2^ = 0.999 and 0.996, respectively. The intensity of 5-HMFA and of FA peaked on the fitting curves was at 1.34×10^3^ µV and 2.06×10^3^ µV ng^−1^ of the authentic compound, respectively. The absolute detection limits of 5-HMFA and FA in 10-µL samples (from the volumetric solution) were both 0.1 ng, and all the samples contained 5-HMFA and FA in the range of 0.7 to 15 ng, which was within the calibration circuit for the standard curves. All quantitative analyses proceeded in triplicate (three tubes per sample) and the following extraction process was repeated three times.

We quantified 5-HMFA and FA in the MeOH-washed agar powders and in several dry agar products (5.00 g scale) to quantify the remaining traces of the compounds. Gellan gum was also analysed by HPLC. The absolute detection limits of 5-HMFA and FA in 10-µL samples (from the volumetric solution) were both 0.1 ng, and all of the samples contained 5-HMFA and FA in the range of 0.7 to 15 ng, which was within the standard calibration curve. All quantitative analyses were performed in triplicate (three tubes per sample), and the following extraction process was repeated three times: a 5.00 g portion of agar powders in 50-mL Falcon tubes were soaked in 25 mL of MeOH, sonicated for 30 min, centrifuged (3,500×*g*), and recovered. This process was repeated three times for a calculated recovery of 99.5%. The combined MeOH extracts were dehydrated, dissolved in 1 mL of Milli-Q water, filtered through 0.45-µm PTFE membranes, and directly applied to Sep-Pak C_18_ column cartridges (Varian). The filtrates were collected in 5-mL volumetric flasks. A small portion of this volumetric solution was filtered through a 0.22-µm PTFE membrane, and then 10-µL samples collected using 25-µL Hamilton (N702) syringes were analyzed by HPLC and monitored by UV absorbance at 245 nm. The absolute amount of each compound in 10 µL of the injection volume ranged from 0.1 to 10 ng, with the zero intercept at 10 in the dilution series. All quantitative analyses using the 5-HMFA and FA calibration curves were performed in triplicate (three tubes per sample). Bar, ± standard deviation (n = 3).

### Colony Development of Slow-growing Bacteria by Swarming Acceleration

Three hundred milligrams of tundra soil from the O horizon (organic layer) of Lapland, Finland was inoculated onto gellan gum soft gel medium for an acetylene reduction assay. The culture was diluted 200-fold with water and spread over MW plates containing 1.5% gellan gum. A single colony of the slow-growing actinobacterium *Cryocola antiquus*, which appeared to be one of the most represented cell populations on the plate, was isolated by means of a stroke culture onto another 1.5% gellan gum plate. Several colonies of the plate incubated for 10-day preculturing at 15°C were then suspended in 200 µL of sterile water and diluted to 1×10^2^ cells. A 50-µL aliquot of the suspended cells was spread with a glass spreader onto plates of nitrogen-free Winogradsky’s medium with a 0.05% carbon source mixture (D-fructose, D-glucose, sucrose, D-mannitol, succinic acid and DL-malic acid at molar ratios of 2∶2: 2∶2: 1∶1, modified from a recipe described previously [37]) that had been solidified with 1.5% unwashed agar, 1.5% washed agar, or 1.5% washed agar supplemented with the mixture of furan-2-carboxylic acids equivalent to the original concentrations in 1.5% unwashed agar. All of the plates were incubated at 20°C for 3 weeks, and the colonies that appeared on each plate were photographed alongside 1.5% gellan plates.

All necessary permits were obtained for the described field studies at Kilpisjärvi Biological Research Station, Finland. The permission for the soil sampling was obtained from the Mets Hallitus (the State Forest Enterprise of Finland). Our soil samplings were as follows: using GPS, the sampling points on a transection to dig hole of 20 cm×20 cm at most up to 30 cm depth not to disturb vegetation as much as possible, and took 10 to 50 g of soils at each horizon (O, A, E or B). We did not enter Strict Nature Reserve Areas or disturb endangered or protected species.

## Supporting Information

Figure S1 **Preparation of authentic compounds 5-HMFA and FA and the establishment of calibration curves.** Absolute calibration curve method was used to draw their standard curves. For each concentration, measurement was done in triplicates. Bar, ± SD (n = 3).
(TIFF)

Scheme S1 **Isolation process of 5-HMFA and FA from agar powders.**
(TIFF)

Scheme S2 **Chemical derivatization of ethyl 5-chloromethyl-2- furancarboxylate to obtain 5-HMFA.**
(TIFF)
