# Supplementary figures and images for: Trace Amounts of Furan-2-Carboxylic Acids Determine the Quality of Solid Agar Plates for Bacterial Culture
Source: PLoS One. 2012 Jul 27;7(7):e41142. doi: 10.1371/journal.pone.0041142 (PMC3407156; doi:10.1371/journal.pone.0041142)

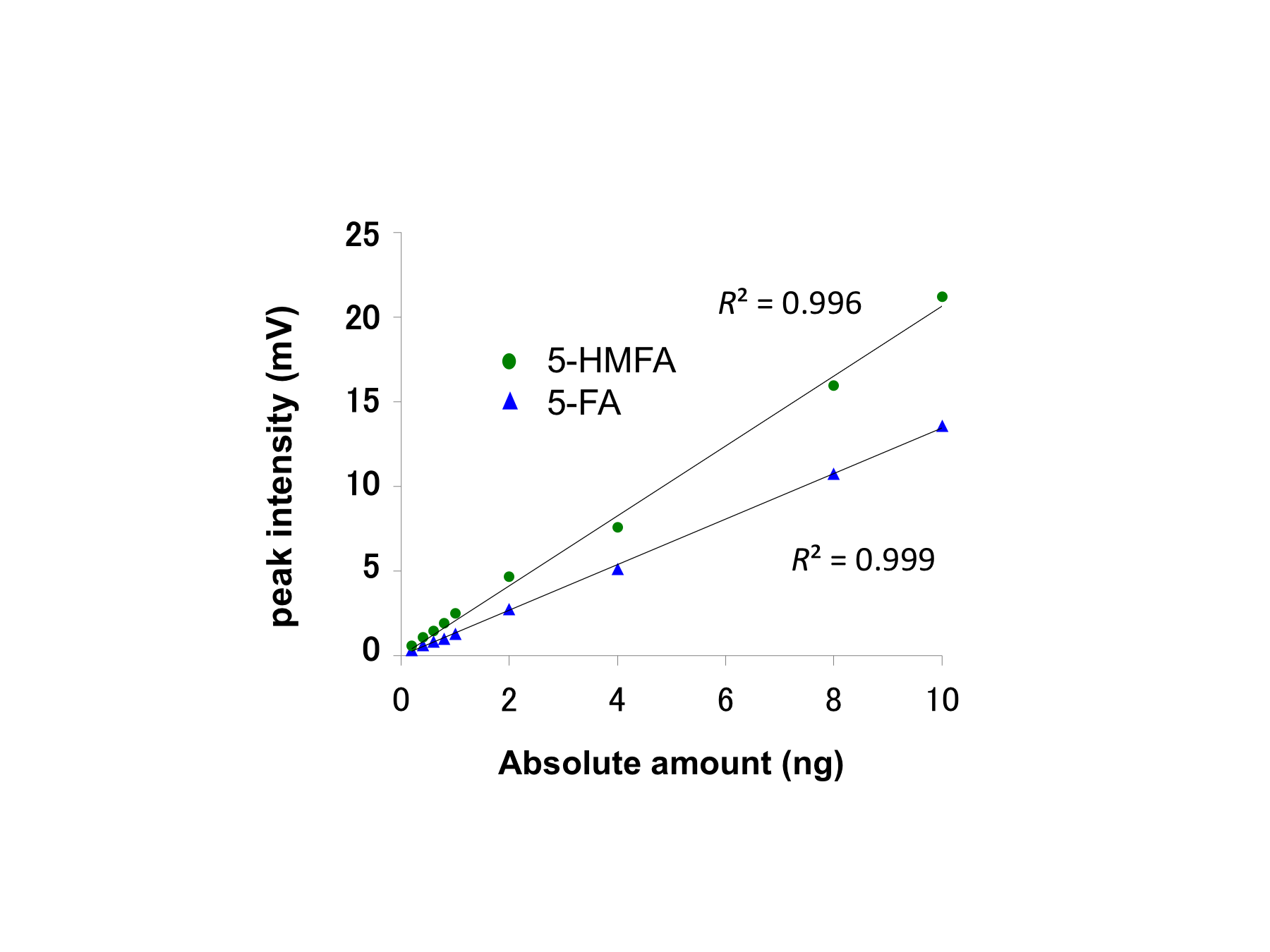

Supplement: Figure S1 — Preparation of authentic compounds 5-HMFA and FA and the establishment of calibration curves. Absolute calibration curve method was used to draw their standard curves. For each concentration, measurement was done in triplicates. Bar, ± SD (n = 3). (TIFF) [file pone.0041142.s001.tiff]

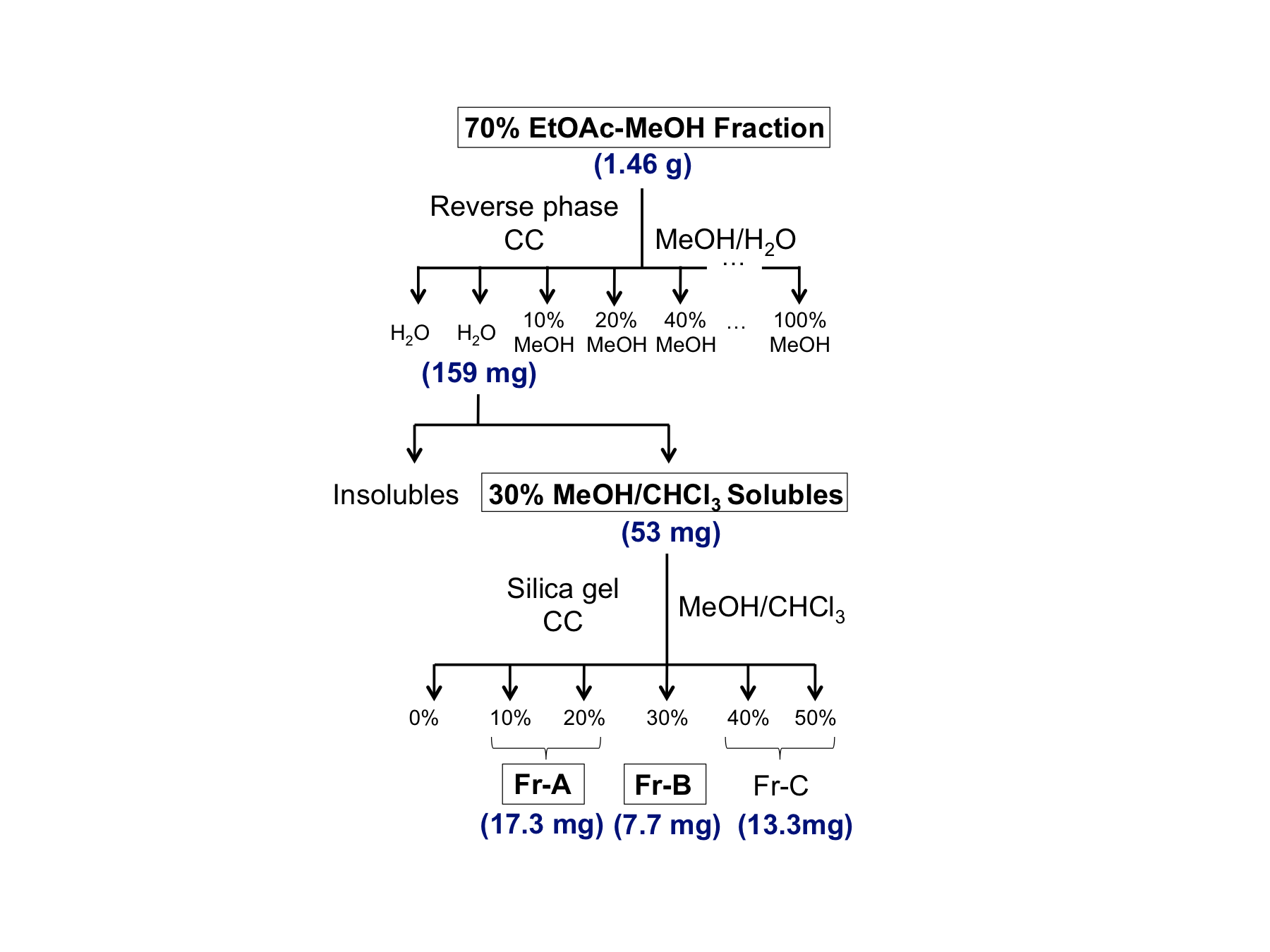

Supplement: Scheme S1 — Isolation process of 5-HMFA and FA from agar powders. (TIFF) [file pone.0041142.s002.tiff]
